# An unusual occurrence of synovial sarcoma in forearm: a case report

**DOI:** 10.11604/pamj.2021.40.187.32269

**Published:** 2021-11-26

**Authors:** Karthikeyan Selvaraj, Bharathiraja Kuppusami, Manimaran Ramachandran, Rajiv Kannan, Ravishankar Kalambur Sundaram

**Affiliations:** 1 Department of General Surgery, Sree Balaji Medical College and Hospital, Chennai, Tamil Nadu, India

**Keywords:** Soft tissue sarcomas, extremities, synovial sarcoma, triceps muscle, case report

## Abstract

Soft tissue sarcomas are group of malignant tumours arising from extra skeletal mesenchymal tissue. Presenting a patient with swelling over the posterior aspect of left arm for 6 months, gradually increasing in size for four months and rapidly increasing in size for last 2 months and not associated with pain. Peripheral pulses felt. Ultrasound showed large solid cystic intramuscular lesion arising from triceps muscle and Trucut biopsy showed poorly differentiated malignancy. Magnetic resonance imaging (MRI) left arm impression was large lobulated solid cystic space occupying lesion in triceps muscle, possibility of neoplastic lesion. Here the patient underwent wide local excision and reconstruction procedure followed by Radiotherapy, chemotherapy and regular follow up.

## Introduction

Soft tissue sarcomas are group of malignant tumours arising from extra skeletal mesenchymal tissues. Most of soft tissue sarcomas are located in lower extremities and next to it 25% [1]. Located in upper extremities and torso followed by hand and neck region. Compared to the lower extremity, where liposarcoma and myxoid sarcoma are commonly encountered; synovial sarcoma, epithelioid sarcoma, and fibro-sarcoma are relatively more common in the upper extremity [2,3]. There is slight male predominance. Gold standard treatment of soft tissue sarcoma is surgical resection with adequate margin clearance.

## Patient and observation

A 28-year-old male patient came with complaints of swelling over the posterior aspect of left arm for 6 months. The swelling was initially small in size insidious in onset, gradual increasing in size for four months and rapidly increasing in size for last 2 months and not associated with pain. No history of trauma, loss of weight and any other specific complaints. Nil significant family history, No similar complaints in family members, No history of any other swellings in the body. No co morbidities for this patient, No similar complaints in past.

**Figure 1:**
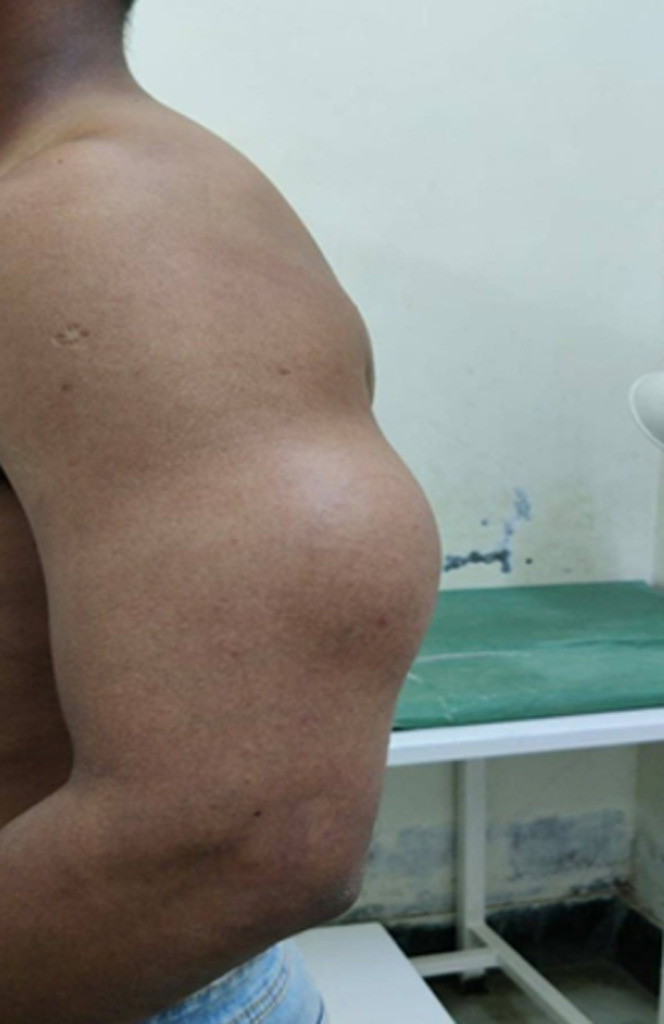
pre-operative image

**Clinical findings:** physical examination revealed (Figure 1) a single swelling of size 15×10 cm present over the posterior aspect of Left arm, 20cm inferior to the Left acromion process and 15cm inferior to the Left olecranon process smooth in surface, hard in consistency and swelling- mobility restricted on triceps muscle contraction. No pulsation felt over the swelling. Left axillary, left brachial and left radial pulses felt. No regional lymph nodes.

**Figure 2:**
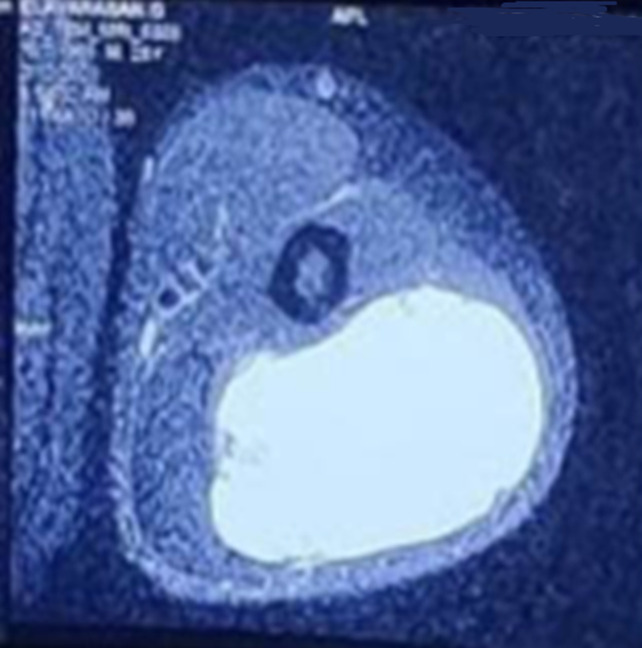
MRI left arm showing large lobulated solid cystic space

**Diagnostic assessment:** all routine investigations were done and it was within normal limits. Ultrasound showed large solid cystic intramuscular lesion in the posterior aspect of left arm, possibly arising from triceps muscle and Trucut biopsy showed poorly differentiated malignancy. MRI left arm impression was large lobulated solid cystic space occupying lesion of size 9.5cm×5.6cm ×5.4cm intramuscularly in the triceps muscle-possibility of neoplastic lesion (Figure 2).

**Diagnosis:** synovial sarcoma - high grade - stage 3 with unfavorable prognosis.

**Figure 3:**
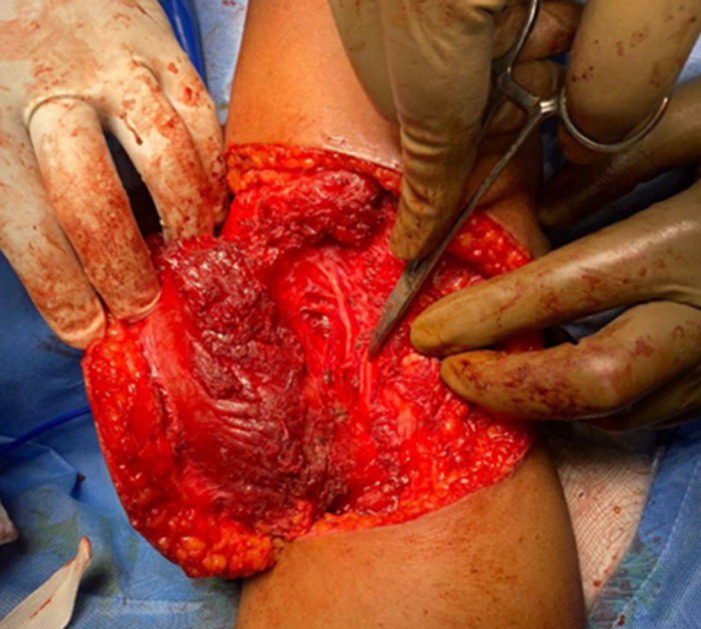
intra-operative picture showing median nerve

**Figure 4:**
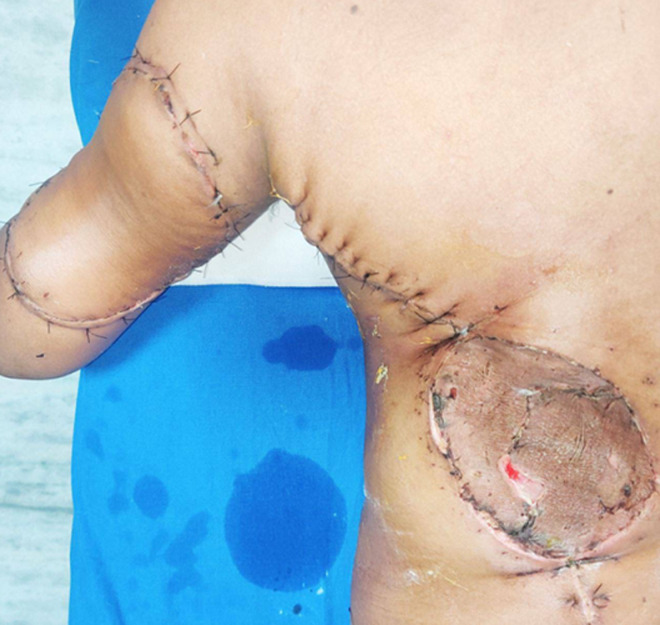
post-operative image

**Therapeutic intervention:** after obtaining cardiology and anaethetic fitness patient was preceded for wide local excision of triceps muscle with left lattismus dorsi flap reconstruction with split skin grafting of the raw area of the back (Figure 3). Intra-operative specimen was too sent for histopathological examination and immune-histochemistry. Postoperative period was uneventful. Patient was given adequate analgesics, antibiotics and all supportive measures. Graft was healthy (Figure 4).

**Figure 5:**
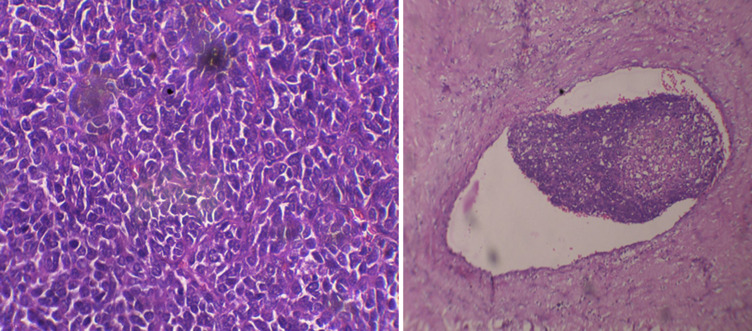
histological section showing embryonal rhabdomyosarcoma

**Follow up and outcome of interventions:** physiotherapy was given simultaneously. Patient was able to move his limb in all directions. Histopathology report showed high grade sarcoma, histomorphology favoring embryonal rhabdomyosarcoma and immunhistochemistry showed vimentin and Tle -1 positive which concluded with synovial cell sarcoma (Figure 5). Patient was further started with radiotherapy and chemotherapy.

**Patient perspective:** I have been throughout examined by my doctor and have been updated about my condition and the surgery to be done and the outcomes briefly in my native language and reassured.

**Informed consent:** yes, patient was informed about his condition and the surgical procedure performed on him and the outcome of the procedure in his native language. It has been also discussed with his family members and they also consented to the procedure.

## Discussion

The most well-known site of soft tissue sarcoma is at the lower furthest point (60%), followed by the furthest point (23%) (with inclination for the shoulder), and the head and neck (9%). Further, it will in general be found near the huge joints of the limits, particularly the knee and lower leg.

### Clinical management

**Surgery:** the gold standard treatments of sarcomas are complete surgical resection. The primary goal of the procedure is enbloc resection of tumour through normal tissue planes and should include the previous incision biopsy scar. As the synovial sarcoma usually encases the neurovascular bundles, injury to these structures can be prevented by adequate exposure and meticulous dissection. For recurrent tumours, surgery is the gold standard option. As majority of patients undergo limb sparing surgery and amputation is still considered as a final resort, amputation rate is still elevated [4]. For patients with recurrence, new adjuvant systemic therapy is the treatment of choice [2]. Careful patient selection is necessary for the treatment of metastatic synovial sarcoma, based on their response [5]. Synovial sarcomas are a high grade tumour, which responds to adjuvant radiation therapy in the form of external beam radiation, brachytherapy and intensity modulated radiotherapy. For metastatic disease, chemotherapeutic agent Ifosfamide is the treatment of choice. Various modalities of treatment have been implemented to improve the survival of patients with high risk tumours [4]. Another modality of treatment that seems to provide promising results is epidermal growth factor family.

## Conclusion

Synovial sarcomas are the most common malignant tumours usually less than 5cm in size. They usually present early and distant metastasis is rare. Diagnosis made depending on the lesion which is calcified not anywhere near the joint in a young individual. Cross-sectional imaging studies helps in careful dissection. The small size of tumour and its slow growing nature attributes to misdiagnosis. Since synovial sarcomas are high grade tumours, adequate margin clearance is essential to prevent recurrences.
